# Combined Metabolome and Transcriptome Analyses of Young, Mature, and Old Rhizome Tissues of *Zingiber officinale* Roscoe

**DOI:** 10.3389/fgene.2021.795201

**Published:** 2021-12-08

**Authors:** Huanfang Liu, Honghua Yang, Tong Zhao, Canjia Lin, Yongqing Li, Xinhua Zhang, Yushi Ye, Jingping Liao

**Affiliations:** ^1^ Guangdong Provincial Key Laboratory of Applied Botany, South China Botanical Garden, Chinese Academy of Sciences, Guangzhou, China; ^2^ College of Biological and Brewing Engineering, Taishan University, Taian, China; ^3^ Guangdong Eco-Engineering Polytechnic, Guangzhou, China; ^4^ College of Life Sciences, University of Chinese Academy of Sciences, Beijing, China

**Keywords:** diarylheptanoid, 6-gingerol, ginger rhizome growth, HiSeq Illumina sequencing, linoleic acid metabolism, phytohormone signaling, starch and sucrose, UPLC-MS/MS

## Abstract

Ginger (*Zingiber officinale* Roscoe) is known for its unique pungent taste and useability in traditional Chinese medicine. The main compounds in ginger rhizome can be classified as gingerols, diarylheptanoids, and volatile oils. The composition and concentrations of the bioactive compounds in ginger rhizome might vary according to the age of the rhizome. In this regard, the knowledge on the transcriptomic signatures and accumulation of metabolites in young (Y), mature (M), and old (O) ginger rhizomes is scarce. This study used HiSeq Illumina Sequencing and UPLC-MS/MS analyses to delineate how the expression of key genes changes in Y, M, and O ginger rhizome tissues and how it affects the accumulation of metabolites in key pathways. The transcriptome sequencing identified 238,157 genes of which 13,976, 11,243, and 24,498 were differentially expressed (DEGs) in Y vs. M, M vs. O, and Y vs. O, respectively. These DEGs were significantly enriched in stilbenoid, diarylheptanoid, and gingerol biosynthesis, phenylpropanoid biosynthesis, plant-hormone signal transduction, starch and sucrose metabolism, linoleic acid metabolism, and α-linoleic acid metabolism pathways. The metabolome profiling identified 661 metabolites of which 311, 386, and 296 metabolites were differentially accumulated in Y vs. M, Y vs. O, and M vs. O, respectively. These metabolites were also enriched in the pathways mentioned above. The DEGs and DAMs enrichment showed that the gingerol content is higher in Y rhizome, whereas the Y, M, and O tissues differ in linoleic and α-linoleic acid accumulation. Similarly, the starch and sucrose metabolism pathway is variably regulated in Y, M, and O rhizome tissues. Our results showed that ginger rhizome growth slows down (Y > M > O) probably due to changes in phytohormone signaling. Young ginger rhizome is the most transcriptionally and metabolically active tissue as compared to M and O. The transitioning from Y to M and O affects the gingerol, sugars, linoleic acid, and α-linoleic acid concentrations and related gene expressions.

## Introduction

Ginger (*Zingiber officinale* Roscoe) is a perennial herb that belongs to the Zingiberaceae family. It originated in Southeast Asia and then spread to other parts of the world owing to its unique taste and medicinal uses (Ai et al., 2005). In China, it is being produced for more than 2,000 years and is an important herb in traditional Chinese medicine. China ranks second after India in ginger production; China produced 552,192 metric tons of ginger in 2019 (www.nationamaster.com). Prime uses of ginger are food and pharmaceutical industries. Its consumption is usually due to the health beneficial activities of different metabolites. The health benefits include neuroprotective and cognitive-enhancing effects, anti-nausea, anti-inflammatory and antioxidant effects, anti-microbial, and anti-cancer effects (Singletary, 2010; Ma et al., 2021). The unique flavor of the ginger is due to a group of volatile phenolic compounds known as gingerols (Chen et al., 2020a). These gingerols (mainly 6-gingerol) have been reported to restore the damaged intestinal barrier functions, it also prevents oxidative damage to the colon, and is used in the prevention of chronic ulcerative colitis (de Lima et al., 2018; Ma et al., 2021). Other than gingerols, the other two important bioactive compound classes are diarylheptanoids and volatile oils (Jiang et al., 2006). The volatile oil is mainly composed of sesquiterpenoids and monoterpenoids whereas diarylheptanoids belong to a class of derivatives having a 1,7-diarylheptane skeleton e.g., curcuminoids (Jiang et al., 2007). Together, these three compound classes contribute to the overall taste and health benefits of ginger. In addition, ginger is also rich in flavonoids, which too have antioxidant potential and help to lower the risks associated with cancer, blood pressure, and heart disease (Knekt et al., 2002).

Understanding the molecular basis and genetic control of the bioactive compounds present in ginger is an active topic. The prime compound i.e., 6-gingerol is biosynthesized through stilbenoid, diarylhepatnoid, and gingerol biosynthesis pathway (Jiang et al., 2018). The biosynthesis of 6-gingerol involves several important enzymes such as caffeoyl-CoA-O-methyltransferase (CCoAOMT), 4-coumarate CoA ligase (4CL), cinnamate 4-hydroxylase (C4H), and phenylalanine ammonia lyase (PAL). These enzymes are also involved in the biosynthesis of other secondary metabolites in phenylpropanoid biosynthesis pathway (Koo et al., 2013; Cheng et al., 2018; Qian et al., 2019), as this pathway is present upstream of the gingerol biosynthesis pathway. Earlier studies have reported that the concentration of the bioactive compounds vary in the different processed ginger rhizomes i.e., dried, carbonized, stir-fried, and fresh rhizomes (Li et al., 2016). The antioxidant potential of the differently processed gingers varied according to the content of the bioactive compounds of the ginger (Li et al., 2016). However, it is yet to be explored that what parts of the freshly harvested ginger rhizome contain the higher concentrations of the bioactive compounds. In this regard, the changes in the expression of the key genes in major pathways followed by the differential accumulation of the bioactive compounds can be an ideal tool.

Earlier attempts using transcriptome analysis have tried to explore the genetic basis underlying the biosynthesis of volatile oil, gingerols, and diarylheptanoids in young (Y) and mature (M) rhizome tissues (Li et al., 2020). However, the changes in the expression of the relevant genes of the gingerol biosynthesis pathway, as well as the upstream pathways in old (O) rhizome tissues have not been discovered yet in comparison to Y and M rhizome tissues. Furthermore, there is no information on the content variation of these metabolites in Y, M, and O rhizome tissues. Recent advancements in transcriptome sequencing and metabolome profiling have geared up the discovery and biosynthetic pathways of different compounds in medicinal plants (Gaur et al., 2016). For example, an integrated analysis of transcriptome and metabolome analysis of different parts of *Lindera aggregata* provided insights into the potential applications of the compounds from different plant parts and their analgesic effects (Peng et al., 2020). Similarly, this approach helped in the characterization of alkaloids and flavonoids in *Sophora flavescens* (Wei et al., 2021) and *Carthamus tinctorius* L. (Chen et al., 2020b). Another study adapted the coupling of transcriptome-metabolome together with phylogenetic analysis to dissect the polyphyllins biosynthesis pathway in an endangered herbaceous plant *Paris polyphylla* var. *yunnanensis* (Song et al., 2021)*.*

To our knowledge, this is the first attempt that used a combined approach (transcriptome and metabolome) to study the differential variations in the expression profiles and metabolite accumulation in Y, M, and O tissues of the ginger rhizome. We explored the differentially expressed genes (DEGs) and differentially accumulated metabolites (DAMs) enriched in stilbenoid, diarylhepatnoid, and gingerol biosynthesis, plant-hormone signaling, starch and sucrose metabolism, phenylpropanoid biosynthesis, linoleic acid metabolism, and α-linoleic acid metabolism pathways.

## Results

### RNA Sequencing of Ginger Tissues

**FIGURE 1:**
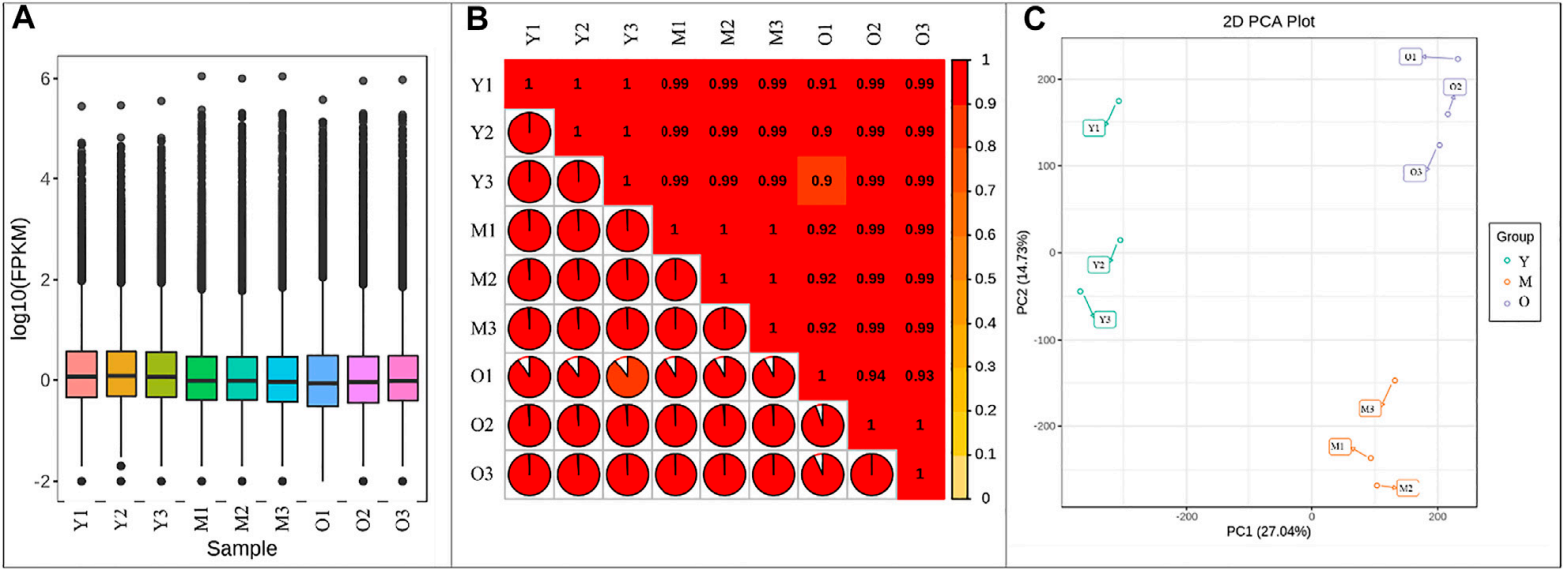
**(A)** Overall distribution of gene expression in young (Y), mature (M), and old (O) ginger tissues. **(B)** Pearson’s Correlation Coefficient, and **(C)** Principal Component Analysis of the gene expression in ginger tissues. 1, 2, and 3 with the sample names represent the replicates.

Illumina sequencing of libraries for the nine ginger samples resulted in 41.27–47.09 million clean reads. A total of 60.15 Gb clean data were obtained; the average clean data of each sample reached 6 Gb. The Q30 base % was 93% and above. The error rate was ≤0.03, GC% ranged between 47.62–50.38 (Supplementary Table S1). Of the total 238,157 genes, 120,876, 116,105, and 135,662 genes could be functionally annotated. 70.61–82.54% reads could be mapped (Supplementary Table S1). Overall fragments per kilobase of transcript per million fragments mapped (FPKM) values of Y tissue were higher than M and O (Figure 1A). The Pearson’s Correlation Coefficients (PCC) between the replicates was higher than 0.9 indicating the reliability of the expression results (Figure 1B). The Principal Component Analysis indicated that the replicates of each treatment were grouped together. The principal component PC1 and PC2 showed 27.04 and 14.73% variability (Figure 1C).

### Differential Gene Expression Between Young, Mature, and Old Ginger Tissues

**FIGURE 2:**
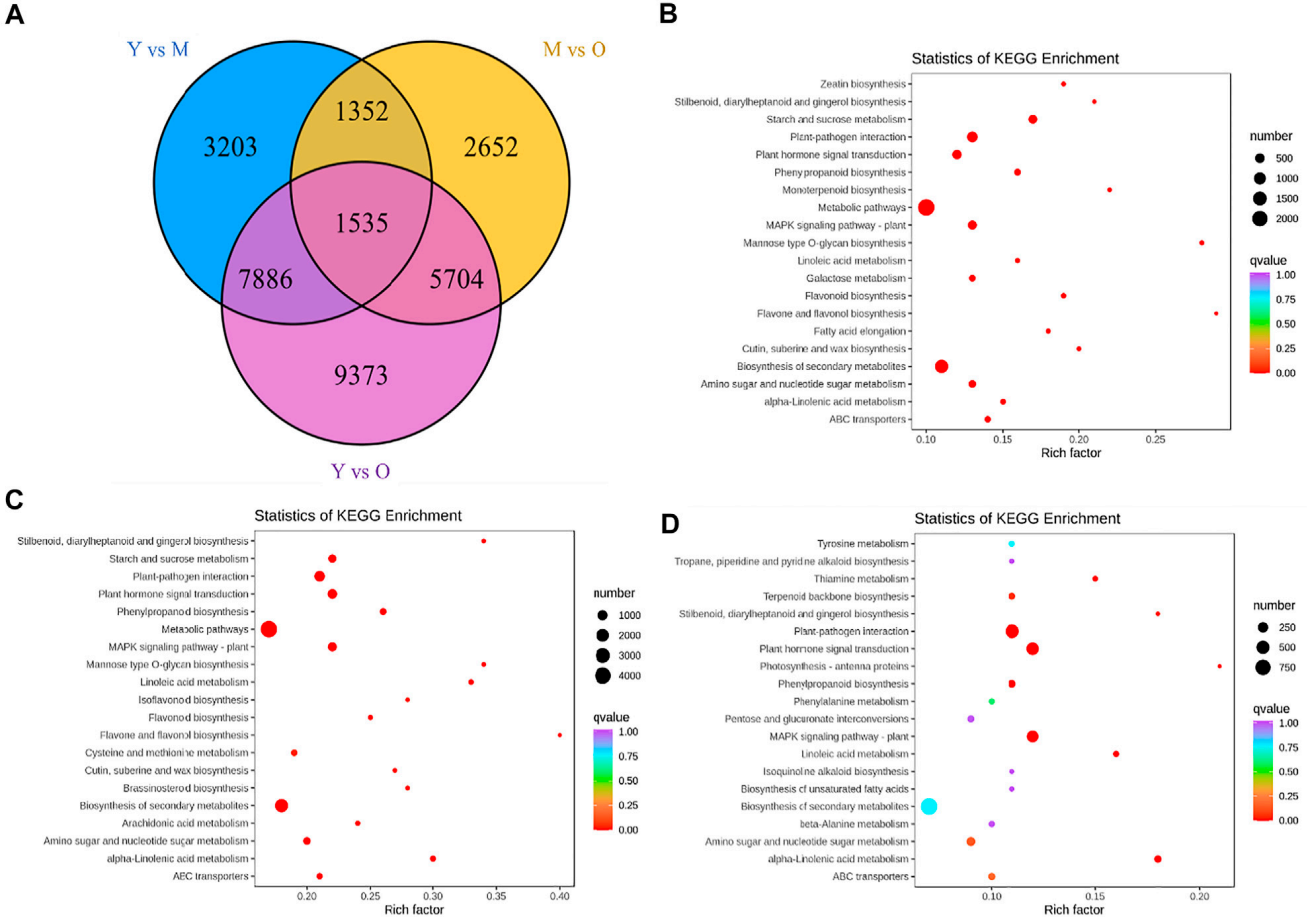
**(A)** Venn diagram representing number of differentially expressed genes (DEGs) specific to each treatment comparison and common DEGs in the three treatment comparisons. KEGG enrichment scatter plots representing pathways to which DEGs were significantly enriched in **(B)** Y vs. M, **(C)** Y vs. O, and **(D)** M vs O.

The screening conditions i.e., |log_2_ fold change| ≥ 1, and false discovery rate (FDR) < 0.05 resulted in the identification of 13,976, 11,243, and 24,498 DEGs in Y vs. M, M vs. O, and Y vs. O, respectively (Figure 2A). Of these, 1,535 DEGs were common in the three treatment comparisons. The KEGG pathway enrichment analysis showed that DEGs were significantly enriched in metabolic pathways, biosynthesis of secondary metabolites, plant-hormone signal transduction, MAPK-signaling pathway, starch and sucrose metabolism, stilbenoid, diarylheptanoid and gingerol biosynthesis, and phenylpropanoid biosynthesis pathway in Y vs. M (Figure 2B). DEGs in Y vs. O were also significantly enriched in these pathways (Figure 2C). In addition to the above-mentioned pathways, we observed that DEGs in M vs. O were enriched in amino sugar and nucleotide sugar metabolism, alpha-linolenic acid metabolism, linolenic metabolism, and terpenoid backbone biosynthesis pathways (Figure 2D). The highest number of DEGs (2029) were enriched in the biosynthesis of the secondary metabolite pathway. We found that a relatively higher number of genes were downregulated in both M and O as compared to Y suggesting that the biosynthesis of secondary metabolites is largely reduced as the ginger rhizome matures or gets older (Supplementary Table S2).

### Differential Regulation of Stilbenoid, Diarylheptanoid and Gingerol Biosynthesis

**FIGURE 3:**
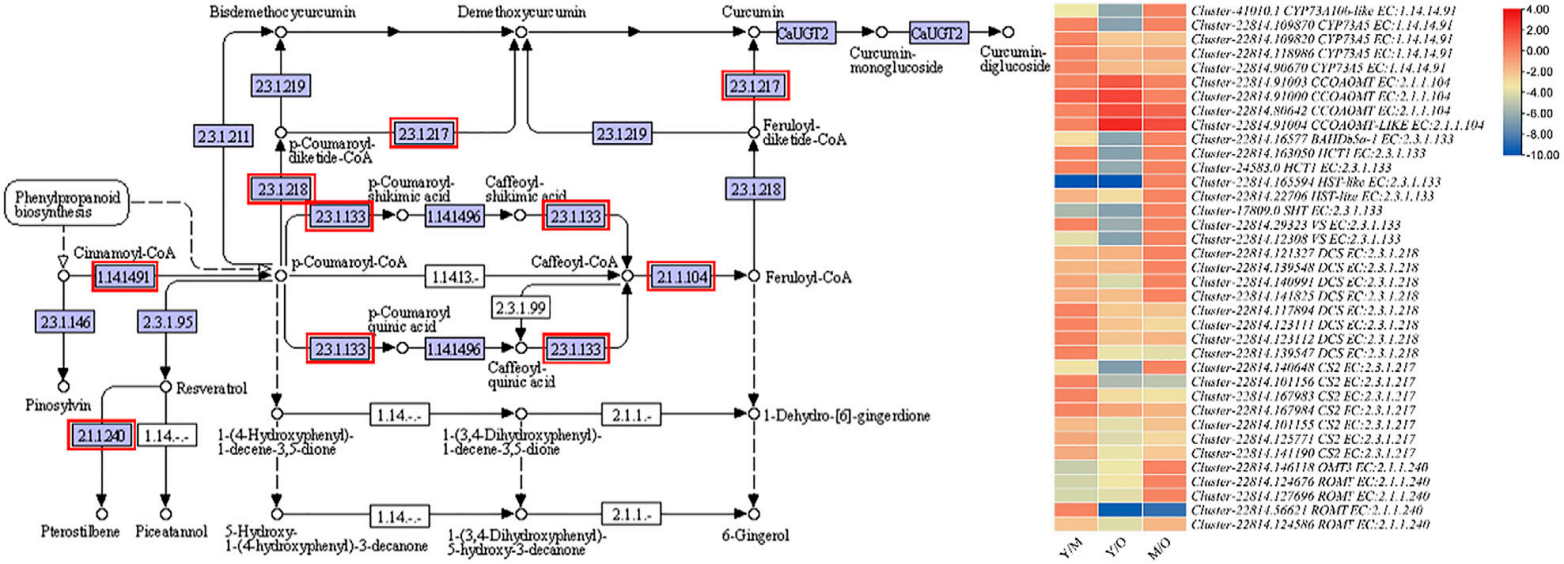
Enrichment of DEGs in stilbenoid, diarylheptanoid and gingerol biosynthesis pathway. The heatmap shows the log2FC values of the DEGs enriched in this pathway. The genes highlighted with red boxes were differentially regulated between the three tissue types i.e., young (Y), mature (M), and old (O).

The most important pathway in the ginger is the stilbenoid, diarylheptanoid, and gingerol biosynthesis because it is the major constituent of essential oil. Furthermore, gingerol contributes to the unique ginger flavor (Jiang et al., 2018). We observed the differential regulation of CYP73A5s (trans-cinnamate 4-monooxygenase-like), CCOAOMT, BAHDb5a-1 (BAHD family acyltransferase), HCT1s (hydroxycinnamoyltransferase 1), HST-like (shikimate O-hydroxycinnamoyltransferase-like), SHT (spermidine hydroxycinnamoyl transferase), VSs (vinorine synthase-like), DCSs (phenylpropanoylacetyl-CoA synthase), CS2 (curcumin synthase 2s), OMT3 (O-methyltransferase 3), and ROMTs (trans-resveratrol di-O-methyltransferase-like) between the three tissue types of the ginger rhizome. Among these, the most important enzymes for the biosynthesis of gingerol is the CYP73As, HCT, HST, SHT, VSs, and CCOAMTs. We observed that the expression of CYP73As decreased with the age of tissues i.e., the highest expression was in Y, followed by M and O. Similar trend was observed in the expression of HCTs, BAHDb5a-1, and SHT. While the expression of HSTs and VSs was variable i.e., the expression of one gene increased while that of the other decreased with the age. Interestingly, we found that the expression of two CCOAMTs (*Cluster-22814.91000* and *Cluster-22814.80642*) increased with the age of tissue, while another gene *Cluster-22814.91003* showed reduction in the FPKM values with the age of tissues. Overall, these expression changes propose that the conversion of Cinnamoyl-CoA to p-Coumaroyl-CoA might decrease as the tissues get older. Similarly, the indirect conversion of p-Coumaroyl-CoA to Caffeoyl-CoA is decreased with the age. While the increasing expression of CCOAMTs suggest that Caffeoyl-CoA conversion to feruloyl-CoA might be increased with the age of tissue, which is either converted to curcumin or 6-gingerol. Since the expression of CS2s decreased with age, it is possible that the increased synthesis of feruloyl-CoA could be directed to 6-gingerol biosynthesis (Supplementary Table S2; Figure 3).

### Differential Regulation of Phenylpropanoid Biosynthesis Pathway

**FIGURE 4:**
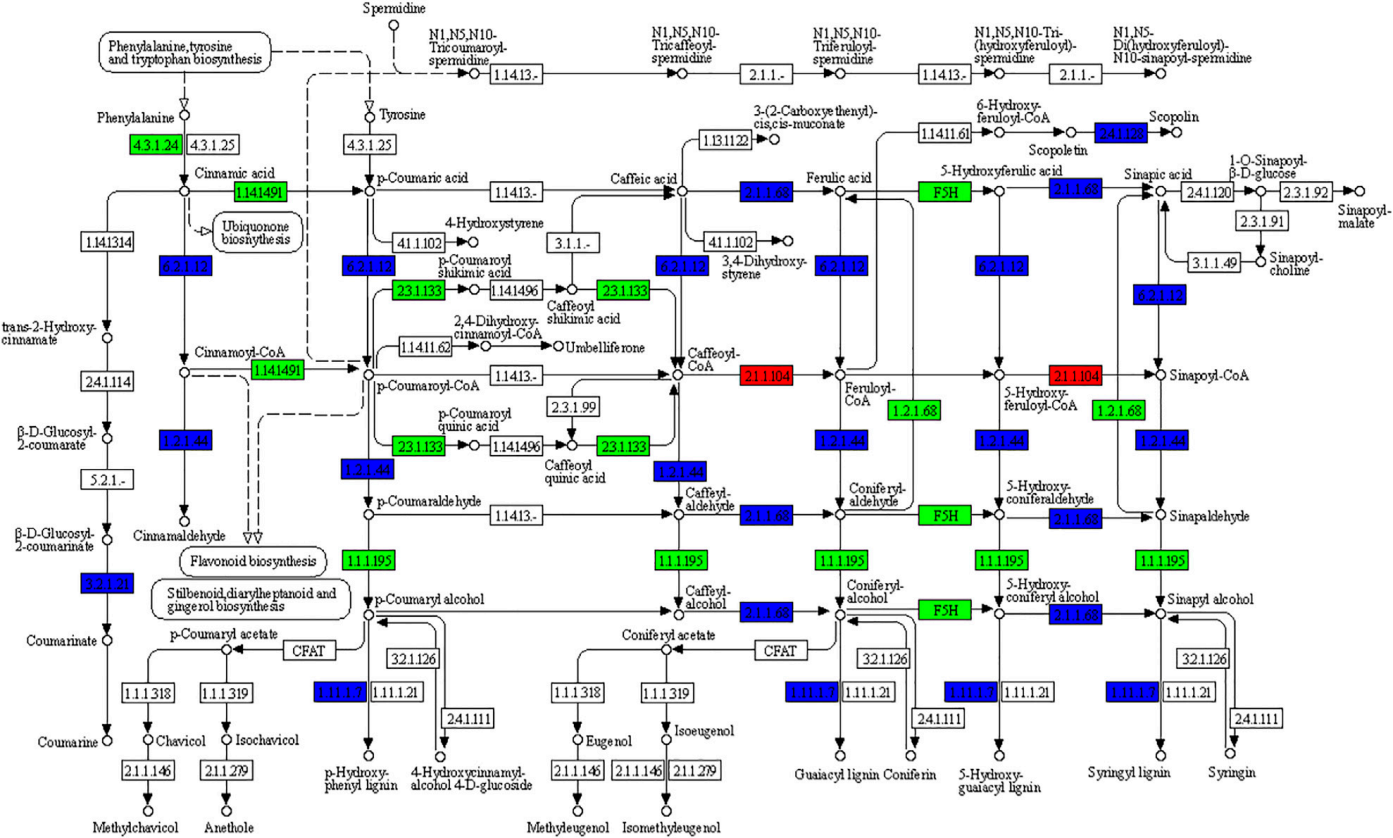
KEGG pathway map representing the differential regulation of phenylpropanoid biosynthesis pathway in Young (Y) vs. Mature (M) ginger rhizome. The numbers in the boxes represent E.C numbers of the enzymes. Red, green, and blue colors represent up-, downregulated, and variably regulated genes between Y and M ginger tissues, respectively. For details on the differential regulation and log2 fold change values, please refer to the Supplementary Table S2.

Phenylpropanoid biosynthesis pathway-derived pharmacologically active metabolites are known to be accumulated in ginger e.g., gingerols (Ramirez-Ahumada et al., 2006). We also observed that phenylpropanoid biosynthesis was among the pathways in which DEGs were significantly enriched (Figure 3); 222 DEGs were enriched in this pathway. The differential regulation of a large number of genes indicates significant changes in the biosynthesis of pharmacologically active compounds between the rhizomes of different ages. Of these 222, 116, 222, and 79 were differentially expressed in Y vs. M, Y vs. O, and Y vs. M, respectively. Only 20 genes were upregulated in M as compared to Y, while 96 were downregulated. Most of the upregulated genes were peroxidases (PODs), beta-glucosidase 22’s (BGLU22s), 4-coumarate--CoA ligase-like 5 (4CL5s), and caffeic acid 3-O-methyltransferase (COMTs). The downregulation of a large number of DEGs in M as compared to Y suggests that the biosynthesis of phenylpropanoids decreased when the ginger tissues get mature (Figure 4). A similar trend was observed when we compared Y with O tissues i.e., only 49 of the 222 genes were upregulated in O as compared to Y. By looking into the DEGs in M vs. O, we noted the upregulation and downregulation of 18 and 60 genes in O as compared to M, respectively (Supplementary Table S2).

### Differential Regulation of Starch and Sucrose Metabolism Pathway

We also observed that a large number of DEGs (483) were regulated between the three tissue types; these genes were annotated as 28 different enzymes in the starch and sucrose biosynthesis pathway (Supplementary Table S2). Of these, 150 genes were associated with starch and sucrose biosynthesis related enzymes. In the Y and M tissue comparison, the biosynthesis of starch/glycogen, amylose, sucrose-6P, D-glucose-6P, and UDP-glucose was possibly increased since the genes i.e., 1,4-alpha-glucan-branching enzyme, granule-bound starch synthase (GBSS), sucrose-phosphate synthases (SPSs), phosphoglucomutase (PGM), and UTP--glucose-1-phosphate uridylyltransferases (UGPs) were upregulated. On the other hand, the conversion of cellulose to cellobiose *via* endoglucanase (EGs) was possibly reduced due to their decreased expression in M as compared to Y. The conversion of trehalose to D-glucose was probably reduced due to the decreased expression of trehalase in M as compared to Y. When we compared the Y vs. O, we observed almost similar trends i.e., the increased expression of genes related to the biosynthesis of sucrose (SPS2-like), amylose (GBSS), and D-glucose (4-alpha-glucanotransferase, DPEs). Similar to Y vs. M, we also noticed the decreased expression of trehalases in O as compared to Y suggesting that the conversion of trehalose to D-glucose was decreased. Interestingly, we noted that the expression of genes related to the biosynthesis of maltose (alpha-glucosidases and beta-amylases) and D-fructose (alpha-glucosidase) was reduced in O as compared to Y (Supplementary Table S2; Supplementary Figure S1). These observations based on the expression changes in the respective genes related to starch and sucrose biosynthesis suggest that the UDP-glucose-6P, D-glucose, sucrose, and starch/glycogen is higher in M and O as compared to Y ginger rhizomes and might differ (increase/decrease) with age.

### Regulation of Plant-Hormone Signal Transduction Pathway in Young, Mature, and Old Ginger Tissues

Recent studies have explained the role of plant hormones in rhizome development in ginger (Chen et al., 2020). We also found that the plant-hormone signal transduction pathway was among the KEGG pathways in which the DEGs were significantly enriched (Figure 3); A total of 791 DEGs were enriched in this pathway (Supplementary Table S2). We found that the genes related to auxin signaling were downregulated in M and O as compared to Y (Supplementary Figure S2). Majorly, we observed that auxin response factors (ARFs) i.e., ARF1, ARF2, ARF3, ARF5, ARF7, ARF11, ARF15, and ARF19, auxin-induced protein (AUX) 22D (AUX22D), AUX-like1, AUX-like2, AUX-like3, and AUX-like4, auxin-responsive protein (IAA) IAA2, IAA6, IAA6-like, IAA16-like, IAA17, IAA17-like, SAUR32, SAUR32-like, SAUR71, and SAUR71-like were differentially regulated between Y and M tissues. Interestingly, only IAAs were downregulated in M as compared to Y, while other genes were both up- and downregulated. On the contrary, we observed that all annotated genes that were enriched in auxin signaling were both up- and downregulated in O as compared to Y. Another important observation was the downregulation of indole-3-acetic acid-amido synthetases (GH1s, GH3.6s, and GH3.8s) in M and O as compared to Y (Supplementary Figure S2).

In the case of genes related to cytokinin signaling, we observed that histidine-containing phosphotransfer protein (AHP) genes were downregulated in M and O as compared to Y. However, their expression changes were not consistent between Y vs. O and M vs. O i.e., they were both up- and downregulated in O as compared to Y while upregulated in O as compared to M. Apart from these expression changes, a major observation was the downregulation of two-component response regulator ARR-A family members in O as compared to M. These expression changes suggest that the cytokinin signaling might not be age specific in ginger rhizomes and the genes might express differently in different aged rhizome tissues. Regarding gibberellin signaling, the gibberellin receptor GID1s were downregulated in M as compared to Y, while all other genes were both up- and downregulated in M. On the other hand, the O ginger rhizome tissues had increased expression of GID2 as compared to Y. Similarly, we observed the higher expression of GID1 in O as compared to M. The genes enriched in abscisic acid (ABA) signaling were both up- and downregulated in M and O as compared to Y. Interestingly, we observed the upregulation of abscisic acid receptor (PYLs) and serine/threonine-protein kinase SAPKs (SAPK). The upregulation of ABA signaling in O as compared to M could lead to dormancy in older rhizomes (Supplementary Table S2; Supplementary Figure S2).

Most of the DEGs that were enriched in other plant hormones’ signaling i.e., ethylene, brassinosteroids, Jasmonic acid (JA), and Salicylic acid (SA) were downregulated in M and O as compared to Y and in O as compared to M. These expression changes propose that as the age of the ginger rhizome increases, the signaling of these hormones become less prevalent. It also proposes that the younger tissues are the most active parts of the rhizome in terms of plant-hormone signaling.

### Differential Regulation of Linoleic Acid and α-Linoleic Acid Biosynthesis Pathways in Young, Mature, and Old Ginger Rhizomes

**FIGURE 5:**
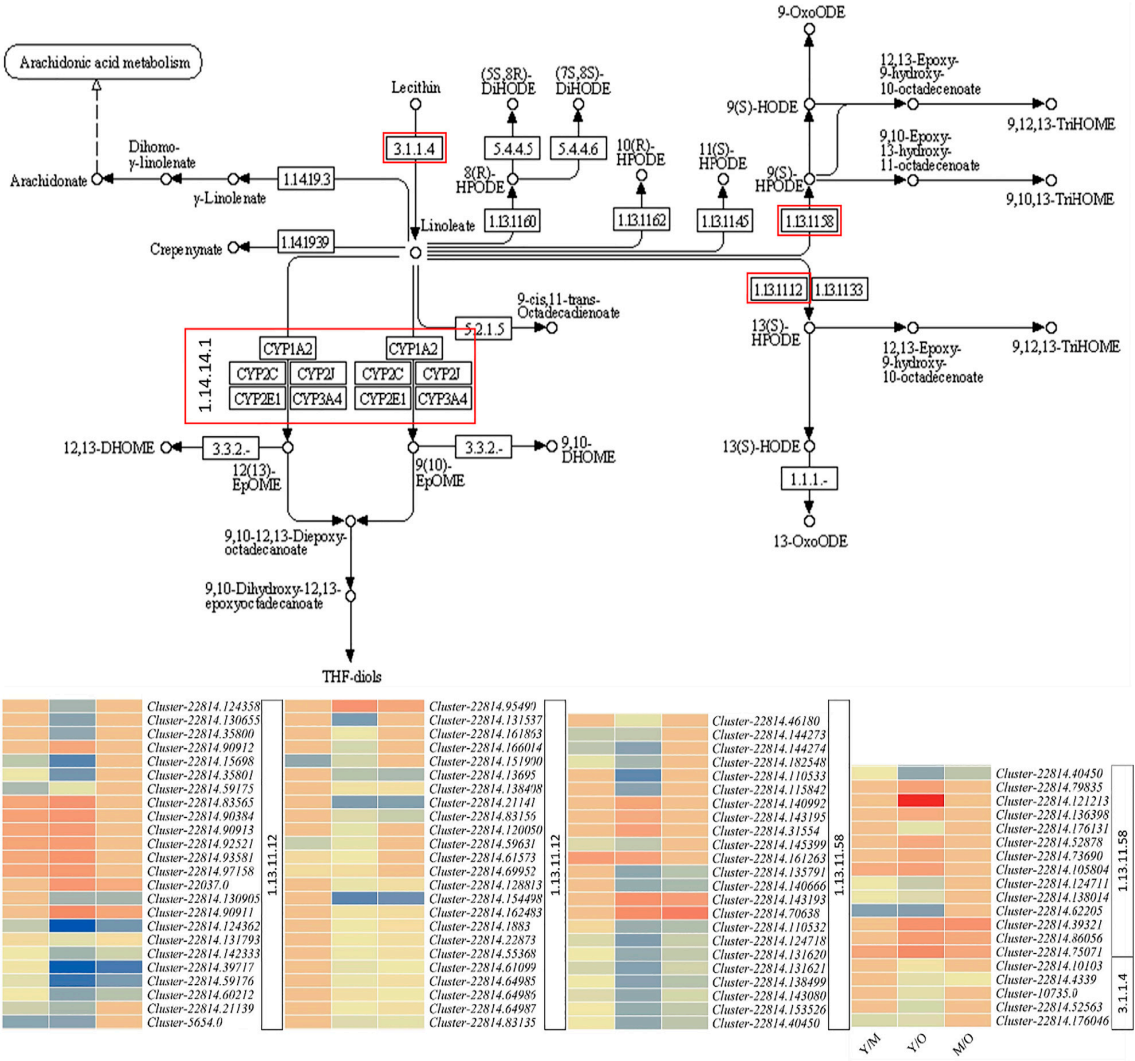
Differential regulation of linoleic acid metabolism pathway in young (Y) mature (M), and old (O) ginger rhizome. The red boxed genes/enzymes were differentially expressed between different tissues. The heatmaps represent the log2 fold change values of the differentially expressed genes. The numbers with heatmaps represent corresponding red boxes in the pathway figure panel i.e., EC: 1.13.11.12 (linoleate 13S-lipoxygenase 2-1, chloroplastic/lipoxygenase), E.C: 1.13.11.58 (linoleate 9S-lipoxygenase), E.C: 1.14.14.1 (cytochrome P450 71A1-like), and E.C: 3.1.1.4 (phospholipase A2-alpha/phospholipase A2 homolog 3-like).

Linoleic acid is a dominant fatty acid in ginger rhizome (Oforma et al., 2019) and is considered beneficial for human health in many ways; it has anti-cancer and anti-inflammatory properties and reduce the risk of atherosclerosis, etc. (Jandacek, 2017). Our results that 90 DEGs were significantly enriched in linoleic acid metabolism show that this pathway is differentially active throughout different aged tissues (Figure 3). We observed that the expression of phospholipase A2-alpha (PLA2α) and its homologs PLA2 homolog 3-like decreased in M as compared to Y. A similar expression pattern of these genes was observed in M vs. O i.e., decreased expression in O as compared to M. Additionally, we observed that the *CYP71A1-like* genes were upregulated in M as compared to Y and in O as compared to M. *CYP71A1-like* genes break down linoleate into fatty acyls (Van Bogaert et al., 2011). Other genes in this pathway were both up- and downregulated in Y as compared to M and O (Figure 5; Supplementary Table S2). These expression changes suggest that the biosynthesis of linoleate in ginger rhizome possibly decreases with the age of tissues and its degradation increases. The same genes i.e., PLA2α and PLA2 homolog 3-like were also enriched in α-linoleic acid metabolism (Figure 5; Supplementary Table S2). This suggests that similar to linoleate, α-linoleic acid biosynthesis might also decrease with the age of the ginger rhizome.

### qRT-PCR Analysis of Selected DEGs

**FIGURE 6:**
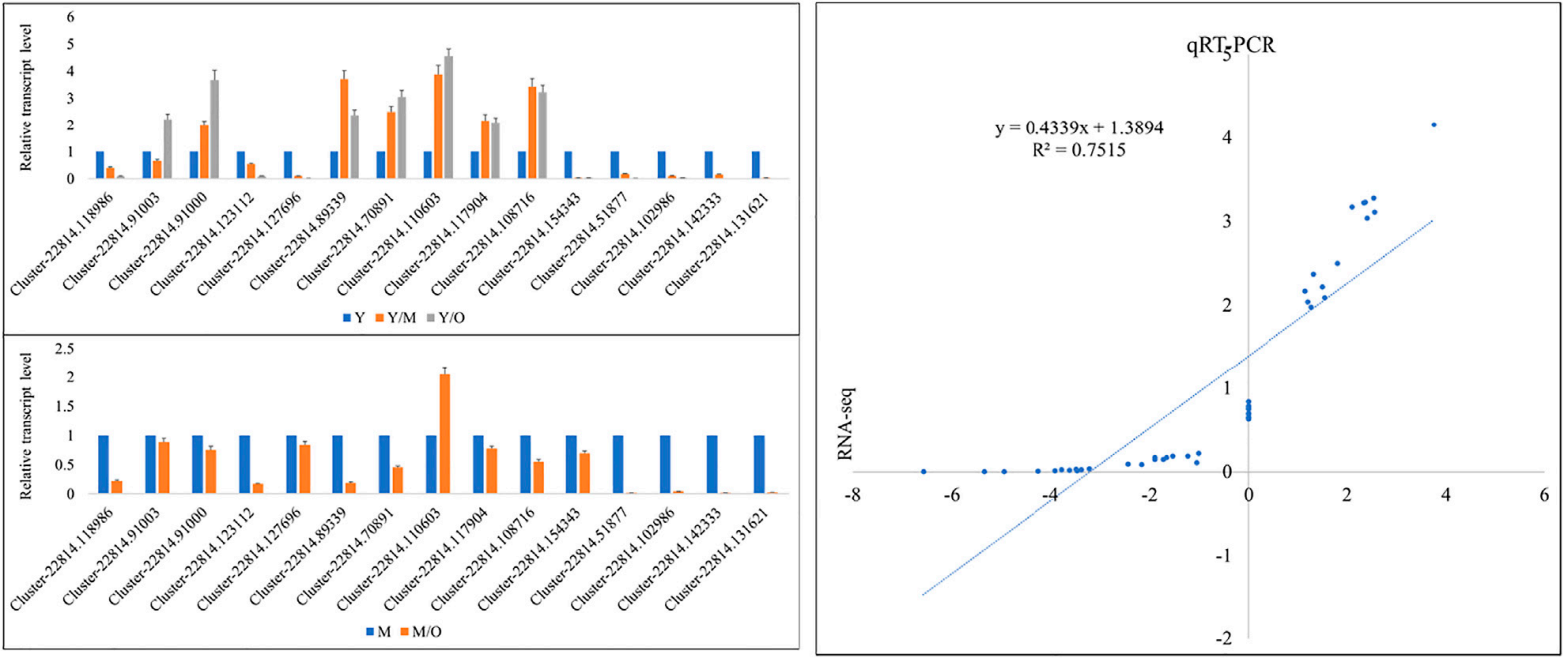
qRT-PCR analysis of selected DEGs in young (Y), mature (M), and old (O) *Z. officinalis* rhizomes. Y/M, Y/O, and M/O shows relative change in the expression. The panel on the right side shows correlation between FPKM values and qRT-PCR data.

The qRT-PCR data for the fifteen ginger genes in Y, M, and O tissues showed similar trend as of the RNA-sequencing data. This was confirmed by the correlation between the FPKM values and the qRT-PCR data i.e., R^2^ = > 0.75 (Figure 6).

### Comparative Metabolomic Profiles of Young, Mature, and Old Ginger Rhizomes

**FIGURE 7:**
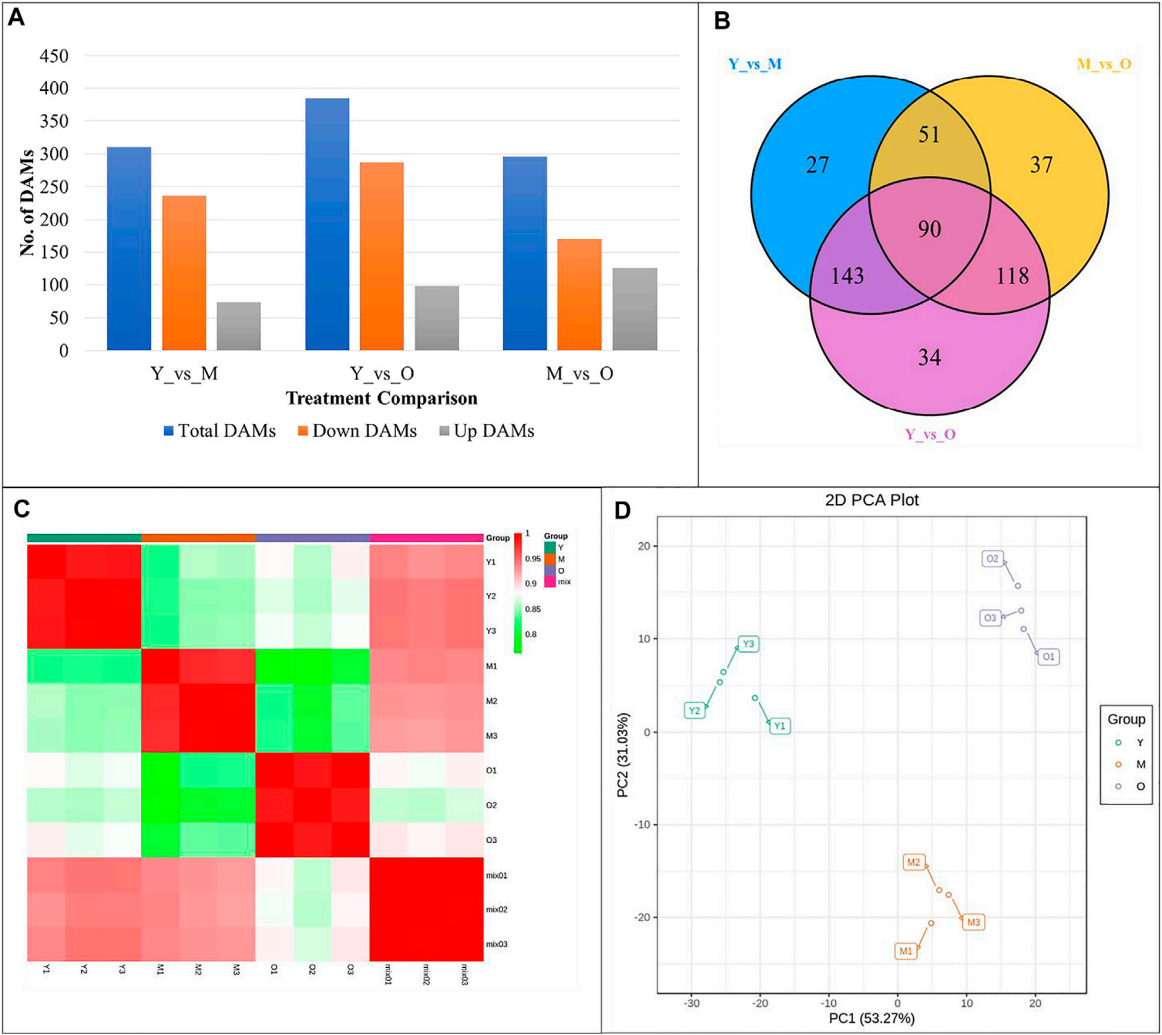
**(A)** Summary, **(B)** Venn diagram, **(C)** Pearson’s Correlation Coefficient, and **(D)** Principal Component Analysis of differentially accumulated metabolites in different ginger rhizome tissues. Y = young, M = mature, O = old ginger rhizome. 1, 2 and 3 with the tissue name represent the replicates.

A total of 661 metabolites were detected in the three tissues of ginger rhizomes based on the UPLC-MS/MS detection platform; 311, 386, and 296 metabolites were differentially accumulated in Y vs. M, Y vs. O, and M vs. O, respectively (Figure 7A). Of the differentially accumulated metabolites (DAMs), 90 were common in all three comparisons (Figure 7B). Principle component analysis indicated that the replicates for each tissue type grouped based on the detected DAMs (Figure 7C) suggesting that there was less degree of variability within the tissue type. The PCC between the replicates ranged between 0.76 and 0.99 with an average of 0.91. This also suggested that the replicated data was reliable (Figure 7D). The differentially accumulated DAMs were classified alkaloids, amino acids and derivatives, flavonoids, lignans and coumarins, lipids, nucleotides and derivatives, organic acids, phenolic acids, quinones, steroids, tannins, and terpenoids.

### Enrichment of DAMs in Different Pathways in Young, Mature, and Old Ginger Rhizomes

Similar to transcriptome sequencing-based KEGG pathway enrichment analyses, we observed that the DAMs were enriched in α-linoleic acid metabolism, linoleic acid metabolism, phenylpropanoid biosynthesis, plant hormone signal transduction, starch and sucrose metabolism, and stilbenoid, diarylheptanoid and gingerol biosynthesis pathways (Table 1).

**TABLE 1:** Log2 Fold change values of differentially accumulated metabolites enriched in different pathways in young vs. mature, young vs. old, and mature vs. old ginger rhizomes.

Met ID	Y/M	Y/O	M/O	Compound	Compound class
Alpha-linolenic acid metabolism					
pmb2786	−1.71	−2.25	0	9-Hydroxy-10,12,15-octadecatrienoic acid	Free fatty acids
mws0367	−1.04	−3.61	−2.57	α-Linolenic acid	Free fatty acids
Linoleic acid metabolism					
pmn001694	0	−3.11	−2.49	9,10,13-Trihydroxy-11-Octadecenoic acid	Free fatty acids
Lmbn003970	0	−3.14	−2.50	9,12,13-TriHOME; 9(S),12(S),13(S)-Trihydroxy-10(E)-octadecenoic acid	Free fatty acids
Rfmb091	−1.75	−1.69	0	9S-Hydroxy-10E,12Z-octadecadienoic acid	Free fatty acids
mws1491	−2.10	−2.76	0	Linoleic acid	Free fatty acids
mws0366	−1.12	−3.88	−2.76	γ-Linolenic acid	Free fatty acids
Phenylpropanoid biosynthesis					
HJN003	0	−1.08	0	1-O-Sinapoyl-D-glucose	Phenolic acids
Lmmn001643	−1.17	−1.00	0	2-Hydroxycinnamic acid	Organic acids
Hmln002806	−1.94	−2.07	0.00	5-O-Caffeoylshikimic acid	Phenolic acids
pmb3074	0	2.54	3.05	5-O-p-Coumaroylquinic acid	Phenolic acids
mws0009	−1.12	−1.26	0	Coniferaldehyde	Phenolic acids
mws0014	0	1.20	0	Ferulic acid	Phenolic acids
pme0021	0	−20.16	−19.85	L-Phenylalanine	Amino acids and derivatives
mws1024	−1.97	−2.50	0	p-Coumaraldehyde	Phenolic acids
mws4085	−1.19	−12.96	−11.77	Sinapic acid	Phenolic acids
pme3443	0	−2.08	−1.27	Sinapinaldehyde	Phenolic acids
mws2212	−1.57	0	1.03	Caffeic acid	Phenolic acids
pme3255	−1.17	0	0	Methyleugenol	Phenolic acids
pme1439	−1.00	0	0	p-Coumaric acid	Phenolic acids
mws0853	−1.24	0	0	Sinapyl alcohol	Phenolic acids
Plant hormone signal transduction					
pme2074	−2.72	−13.57	−10.85	(-)-Jasmonoyl-L-Isoleucine	Organic acids
Lmgn001670	0	0	1.16	Salicylic acid	Phenolic acids
Starch and sucrose metabolism					
pme3313	−1.40	−1.89	0	D-Fructose 6-phosphate	Saccharides and alcohols
mws0866	−1.11	−1.77	0	D-Glucose 6-phosphate	Saccharides and alcohols
mws4170	0	1.53	2.33	D-Glucose	Saccharides and alcohols
pme0519	0	1.41	1.79	D-Sucrose	Saccharides and alcohols
mws0264	0	1.52	1.92	D-Trehalose	Saccharides and alcohols
mws1090	−1.22	−1.77	0	Glucose-1-phosphate	Saccharides and alcohols
Stilbenoid, diarylheptanoid and gingerol biosynthesis					
Hmln002806	−1.94	−2.07	0	5-O-Caffeoylshikimic acid	Phenolic acids
pmb3074	0	2.54	3.05	5-O-p-Coumaroylquinic acid	Phenolic acids
pme2296	0	−2.02	−1.39	Curcumin	Others
mws1560	−1.30	0	0	[6]-Gingerol	Phenolic acids

The important observation was the reduced accumulation of [6]-gingerol biosynthesis in M as compared to Y (log2 fold change = −1.30). However, in the other two comparisons i.e., Y vs. O and M vs. O gingerol was not differentially accumulated. Nevertheless, we observed that 5-O-Caffeoylshikimic acid (present in the upstream of gingerol) accumulation was also reduced in O as compared to Y. Furthermore, since the phenylpropanoid biosynthesis pathway is present upstream of the stilbenoid, diarylheptnoid, and gingerol biosynthesis pathway, the reduced accumulation of most of the phenolic acids in M and O as compared to Y was also observed (Table 1). These observations are in accordance with the transcriptome results and suggest that the gingerol accumulation (and biosynthesis) is possibly reduced when the ginger rhizomes grow older.

Regarding plant hormone signaling, two DAMs i.e., (-)-Jasmonoyl-L-Isoleucine (JA-Ile) and SA were differentially accumulated; JA-Ile accumulation was reduced in both M and O as compared to Y. The change in JA-Ile accumulation might be due to the variation in the accumulation of fatty acids in α-linoleic acid metabolism, which is present upstream of the JA signaling pathway. Regarding α-linoleic acid metabolism and linoleic acid metabolism pathways, we observed that both linoleate and α-linoleic acid were accumulated in lower quantities in M and O as compared to Y ginger rhizome tissues (Table 1). These observations are consistent with the changes in the respective genes’ expression as discussed in the above sections (Supplementary Table S2).

Another important observation was the reduced accumulation of D-fructose-6P, D-glucose-6P, and glucose-1P in M and O as compared to Y ginger rhizome tissues. In the older tissues i.e., O, the accumulation of the D-glucose, D-sucrose, and D-trehalose was increased as compared to Y and M. It could be due to the increased expression of some of the genes associated with this pathway since the transcriptome analysis indicated both up- and downregulation of multiple genes (Supplementary Table S2).

## Discussion

### Young Ginger Rhizome has a High Gingerol Content

Gingerols are the major bioactive component in the ginger rhizome with 6-gingerol being the highly abundant one. It is biosynthesized through stilbenoid, diarylheptanoid, and gingerol biosynthesis pathway (Mao et al., 2019). Our results that the expression of CYP73As, HCTs, BAHDb5a-1, and SHT was higher in Y as compared to M and O indicate that the biosynthesis of 6-gingerol is reduced in M and O as compared to Y (Figure 3). Earlier studies have shown that CYP73A controls phenylpropanoid biosynthesis (cinnamoyl-CoA), which is then converted into [6]-gingerol by downstream reactions (Kim et al., 2021; Mizutani et al., 1997). This is consistent with the observation that the accumulation of [6]-gingerol in M and O ginger rhizomes was reduced as compared to Y (Table 1). This is also explainable due to the lower expression of genes associated with the synthesis of caffeoyl-CoA i.e., HCTs and BAHDb5a-1, and reduced accumulation of 5-O-Caffeoylshikimic acid (Figure 3; Table 1). The expression and relatedness of these genes in gingerol biosynthesis have been described in a recent transcriptome analysis of the ginger rhizome (Jiang et al., 2018). Since the phenylpropanoid biosynthesis pathway is present upstream of gingerol biosynthesis, the changes in the expression of related genes can also be responsible for gingerol content variation in the three studied tissues (Ramirez-Ahumada et al., 2006). The downregulation of a relatively higher number of DEGs in M and O as compared to Y proposes that the biosynthesis of pharmacologically active compounds is reduced in M and O. In this regard, the downregulation of phenylalanine ammonia lyases (PALs), trans-cinnamate 4-monooxygenase-likes (t-C4H-likes) in M and O is important (Supplementary Table S2). These genes convert phenylalanine to cinnamic acid and then to p-coumaric acid, respectively, which is present upstream of the gingerol biosynthesis pathway (Pierrel et al., 1994; Kong, 2015). This is also consistent with the reduced accumulation of L-phenylalanine and p-coumaric acid in M and O (Table 1). Thus, both the expression changes in the phenylpropanoid as well as gingerol biosynthesis pathways and the differential accumulation of metabolites indicate that gingerol biosynthesis reduces in M and O ginger rhizomes.

### Young, Mature, and Old Ginger Rhizomes Differ in Linoleic Acid and α-Linoleic Acid Accumulation

α-linoleic acid is an essential omega-3 fatty acid, which prevents diseases related to heart and blood vessels. Furthermore, it prevents heart attack, reduces blood pressure, and lowers cholesterol (O'Reilly et al., 2020). Linoleic acid plays important role in heart health; studies have shown that when saturated fatty acids are replaced with linoleic acid, it reduces total cholesterol (O'Reilly et al., 2020; De Luis et al., 2011). In this regard, it is important to know which parts of the rhizome have a potentially higher content of the compounds. Our results demonstrated that the accumulation of linoleic acid and α-linoleic acid was higher in Y as compared to O and M (Table 1). This higher content of linoleic acid in Y is probably due to the higher expression of PLA2α and PLA2 homolog 3-like in Y as compared to M and O (Figure 5; Supplementary Table S2). We say this because earlier studies have shown that these genes convert lecithin into linoleate (Burke and Dennis, 2009). The lower content in the M and O tissues could also be due to the breakdown of linoleate into fatty acyls. This proposition is based on the expression changes in *CYP71A1-like* genes; increased expression in M and O as compared to Y indicates a higher breakdown of linoleate (Van Bogaert et al., 2011). Since the PLA2α and PLA2 homolog 3-like also participate in α-linoleic acid biosynthesis from phosphatidylcholine, thus both of these genes are possibly responsible for the decreased accumulation in M and O as compared to Y. All these observations indicate that the fresh growth in the ginger rhizome is richer in α-linoleic acid and linoleic acid contents, thus could be more beneficial when consumed.

### Ginger Rhizome Tissue Growth is Possibly Slowed Down Due to Changes in Phytohormone Signaling

The ginger rhizome’s early growth and maturity is a continuous developmental process and it has been observed that plant hormones’ signaling plays significant roles in such processes (Chen et al., 2020). Furthermore, with maturity, the tissues in rhizomes grow older and the growth processes might be affected by multiple pathways. Our results that the auxin signaling pathway-related genes were downregulated in M and O indicate that the processes such as cell enlargement and plant growth are possibly restricted or less active in M and O as compared to Y. These results are consistent with the previous findings that auxin signaling plays role in plant growth by cell enlargement (Majda and Robert, 2018; Vanderhoef, 1980). In addition to auxins, the cytokinin and gibberellin signaling also play roles in cell division (Zahid et al., 2021; Li et al., 2013). The gibberellins along with ethylene have been recently explored to play roles in the development of rhizomes and rhizome like shoots in oriental Cymbidium hybrids (Zheng et al., 2005). A similar role of these hormone signaling could be expected in Y ginger rhizome since with maturity the rhizome tissues get older and the requirement for growth slows down (Zahid et al., 2021; Whiley, 1980). This is further in line with the lower expression of the genes enriched in brassinosteroids signaling pathway in M and O as compared to Y. Particularly, the differential expression of xyloglucan endotransglucosylase/hydrolase proteins (XETs) and cyclin-D3-2-like genes propose that the processes of cell elongation and cell division are possibly slower in M and O as compared to Y (Supplementary Table S2). This proposition is because XETs are known for brassinosteroid responsive lateral root development (Xu et al., 2020). Similarly, the cyclin-D3-2-like genes cause root cell elongation under different trajectories under the influence of different hormones i.e., brassinosteroids (Takatsuka and Umeda, 2014). Overall, these expression changes suggest that as the ginger rhizome gets mature and older, the cell growth, division, elongation, and development are reduced possibly by the signaling related to auxins, cytokinins, gibberellins, ethylene, and brassinosteroids. These expression changes are important for specific gene characterization and delineating the roles of these phytohormones (and signaling) in ginger rhizome growth.

### Starch and Sucrose Metabolism is Variably Regulated in Ginger Rhizomes

Earlier studies have shown that the starch and sugar contents of fresh ginger increase with growth (Xu et al., 2008; Liu et al., 2020). Our observation that the accumulation of D-glucose, D-trehalose, and D-sucrose was increased in O as compared to Y and M is in accordance with this report (Table 1). This increased accumulation could be linked with the higher expression of GBSS, SPSs, PGM, UGPs, DPEs, and SP2-like since these genes take part in different steps of saccharides and alcohol biosynthesis (Wang et al., 2001; Yuan et al., 2008; Fernie et al., 2001; Schomburg and Stephan, 1997). The observations also propose that there are multiple SPS gene family members that function in the ginger rhizome. Other species such as casava have also been reported to have a multi-gene family of SPSs (Bahaji et al., 2015). The higher content of D-trehalose in M and O might also be due to the changes in the expression of trehalase genes (Supplementary Table S2) since trehalase converts D-trehalose to D-glucose (Elbein et al., 2003). Overall, the accumulation of these saccharides in mature and older tissues is in accordance with the fact that the underground organs of plants i.e., rhizomes and/or rhizomes tend to store higher starch and sucrose contents (Yuan et al., 2008).

Apart from the above discussed pathways, the large number of DEGs and DAMs that were enriched in the biosynthesis of secondary metabolites pathway indicate that in early growth period of the ginger rhizome tissues, the metabolic activity is higher. The larger number of DEGs and DAMs between Y and O indicate that during the transition from younger to older rhizome tissues a lot of metabolic activities are either slowed down or possibly stopped (as evident from a relatively higher number of downregulated genes between Y and O.

## Methods

### Plant Material

**FIGURE 8:**
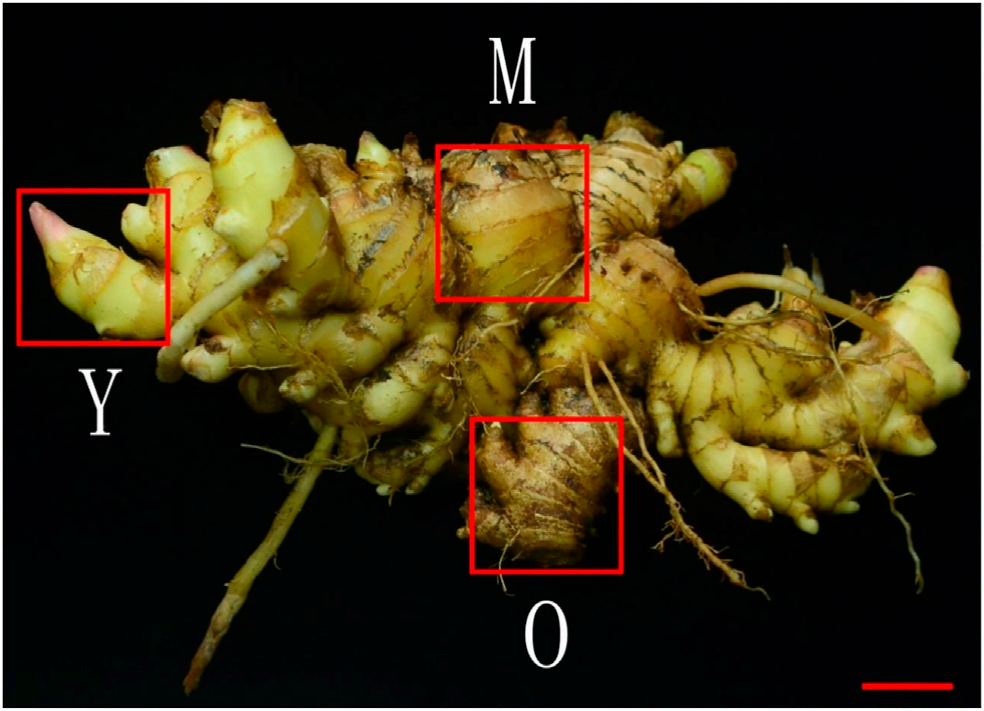
The ginger *Z. officinale* Roscoe, Chinese cultivar Hunan Xiao Jiang. Y (young), M (mature), and O (old) tissues of the rhizome used for transcriptome and metabolome analysis. The red bar indicates 2 cm.

The ginger (*Zingiber officinale* Roscoe) Chinese cultivar Hunan Xiao Jiang was obtained from South China Botanical Garden, Guangzhou, China and the formal identification of this species is performed by the corresponding author of this paper. No voucher specimen has been deposited in an herbarium and no permission is needed to study this species. The plants were cultivated in South China Botanical Garden, Guangzhou, China under natural conditions in March 2020. The climate of the area is humid and the soil type is black. The chosen genotype i.e., Hunan Xiao Jiang is delicate, spicy and has a good taste. Eight months old healthy plants growing on different parts of the field were chosen to prepare three biological replicates. The rhizomes were harvested from each plant and washed well to remove the soil. Three parts from each rhizome i.e., young (Y), mature (M), and old (O) (Fujisawa et al., 2010) (Figure 8) were cut, frozen in liquid nitrogen, and stored at −80°C until the extraction of mRNA and metabolome analysis.

### Transcriptome Analysis

i. RNA Extraction, Library Construction, and Transcriptome Sequencing

Total RNA from the nine replicates were extracted separately by using Spin Column Plant total RNA purification Kit (Sangon Biotech, Shanghai, China) by following the manufacturer’s protocol. The integrity, purity, and concentration of the extracted RNA was determined by using agarose gel electrophoresis, NanoPhotometer spectrophotomer (IMPLEN, Los Angeles, CA, United States), and Qubit 2.0 Fluorometer (Life Technologies, Carlsbad, CA, United States), respectively.

The mRNAs were obtained by enriching them with polyA tails through Oligo (dT) magnetic beads. Following this, we synthesized the cDNA by adding fragmentation buffer to break the mRNA into short fragments. The fragmented mRNAs were used as template to synthesize cDNA with random hexamers by adding buffer, dNTPs, and DNA polymerase I. The cDNA was then purified by using AMPure XP beads (Beckman Coulter, Inc. CA, United States) followed by repairing, A-tailing, and ligation with sequencing adapters. Then the AMPure XP beads were used for fragment size selection. Finally, PCR enrichment was performed to obtain the cDNA libraries.

The quality of the libraries was tested by using Qubit 2.0 for quantification and Agilent 2100 for detection of the library insert size. Furthermore, Q-PCR was used to quantify the effective concentration of the libraries (>2 nM). The libraries were pooled and on-machine sequencing (paired end sequencing in non-standard mode) was performed on Illumina HiSeq platform (Illumina Inc., San Diego, CA, United States).

ii. Bioinformatic Analyses

The sequencing data was filtered to obtain high quality reads by removing the reads with adapters, removing the reads having N content >10%, and removing the paired reads when the number of the low-quality (Q ≤ 20) basis contained in the sequencing reads exceeded 50%.

We then checked the sequencing error rate distribution based on the formulae (Qphred = −10log_10_(e), where e is the sequencing error rate and the base quality value of Illumina is represented by Qphred. Also, we checked the GC content distribution.

BLAST was used to compare the unigene sequences with KEGG (Kanehisa and Goto, 2000), Swiss-Prot (Apweiler, 2001), and GO (Ashburner et al., 2000) databases. Fragments Per Kilobase of transcript per Million fragments mapped (FPKM) values were measured as an index of the expression level of the transcripts/genes. The overall distribution of the gene expression was visually compared by using box plot. The relevance of the biological replicates was determined by measuring the Pearson’s Correlation Coefficient (PCC). Furthermore, the Principal Component Analysis (PCA) was conducted to study the internal structure of the treatments and their relatedness/variability. The differential gene expression between the different tissues was studied by using DESeq2 (Love et al., 2014). The Benjamini-Hochberg method was used to perform multiple hypothesis test correction on the hypothesis test probability (*p* value) to obtain false discovery rate (FDR). The differentially expressed genes (DEGs) were screened if |log_2_ fold change| ≥ 1, and FDR <0.05.

The expression patterns of the DEGs between the Y, M, and O tissues was determined by centralization and standardization of the FPKM values of the genes followed by K-means cluster analysis. Furthermore, the FPKM value were used to do hierarchical cluster analysis. The DEGs comparison between the Y, M, and O were represented by Venn diagrams in InteractiVenn (Heberle et al., 2015). Pathway annotation of the DEGs was done in KEGG (https://www.genome.jp/kegg) (Kanehisa and Goto, 2000) and the DEGs enriched in different pathways were mapped. Scatter charts were developed for the graphical display of the KEGG enrichment analyses results. Finally, the plant transcription factors were predicted in iTAK software (Zheng et al., 2016).

iii. qRT-PCR analysis

15 genes of interest were selected for the qRT-PCR expression analysis. The primers for each gene were designed with the Primer-BLAST tool (https://www.ncbi.nlm.nih.gov/tools/primer-blast/) (Table 2). The *Actin7* gene was used as an internal control. The qRT-PCR reactions were carried out on an ABI Prism 7500 Fast Real-Time PCR System (United States) as reported earlier (Jiang et al., 2018). The qRT-PCR data was analyzed as reported earlier (Livak and Schmittgen, 2001). Correlation between FPKM values and the qRT-PCR expression data was measured in R.

**TABLE 2:** List of primers used for qRT-PCR analysis of the selected ginger genes in different tissues.

Gene ID	Primer forward sequence (5′-3′)	Primer reverse sequence (5′-3′)
*Cluster-22814.118986*	CTAAGTTCCAAGTGT	TGGGTTCAAAAATC
*Cluster-22814.91003*	TCAATTAGAATTGACCTT	TCCGCCAGGCATGGAG
*Cluster-22814.91000*	CATGGTGGCATATCCG	GAATGGCTGCAAGAAAC
*Cluster-22814.123112*	AATGGTACTCAATCATA	GAACGCTTTCTCCTCA
*Cluster-22814.127696*	ATGATGTCATTACC	TTATTTCCTCGGATTCTC
*Cluster-22814.89339*	CATGGTGGCATATCCG	AGAGAAATCAGAATGGA
*Cluster-22814.70891*	TCCTACAATGGAACAAG	TGATGCCTCAGTGTTG
*Cluster-22814.110603*	GAATGGGACCGACAGAA	CTTATCCACGCATGGC
*Cluster-22814.117904*	ATTCATCTAGTTAGAC	GAATGGCTCAATACGGG
*Cluster-22814.108716*	AATGCGGAATCCACAC	CAGGGTTTATGGAGGG
*Cluster-22814.154343*	AGTTCACACTGGCAGT	CATCAACGTCCAGTCTCC
*Cluster-22814.51877*	AGAAATTACCAGACGAGC	ACATTCTGGCTTTGGC
*Cluster-22814.102986*	AGAGGGTTAGGCCTAGTA	CCTATGAATTGGTCTTCC
*Cluster-22814.142333*	GGA​ATG​GAA​CGT​TGC​CTG​A	AACTTTGCACTGGC
*Cluster-22814.131621*	AGTCTACGGCACACTACA	ATAATGGCCGTACCTTTC
*Actin 7*	AGTGTTGGCTTTGTCTAT	AAG​TAG​TCC​CAT​TTG​TTC​T

### Metabolome Analysis

The metabolome analyses were performed as reported earlier (Chen et al., 2019). Detailed methodology is presented below.

i. Sample preparation and extraction

The Y, M, and O tissues of ginger rhizomes were freeze-dried in a vacuum freeze-dryer (Scientz-100F), crushed to powder, dissolved (100 mg crushed sample powder) in 1.2 ml methanol (70%), vortexed for 30 s every 30 min. The process was repeated six times and then the mixtures were placed in refrigerator at 4°C overnight. Next morning, the mixtures were centrifuged at 12,000 × g for 10 min followed by filtration through SCAA-104, 0.22 µm (ANPEL, Shanghai, China) and then the filtrate was used for UPLC-MS/MS analysis.

i. UPLC and ESI-Q TRAP-MS/MS Conditions

UPLC-ESI-MS/MS system (UPLC, SHIMADZU Nexera X2 and MS, Applied Biosystems 4500 Q TRAP) was used for the analyses. The analytical conditions were as follows.

a. UPLC

Column = Agilent SB-C18 (1.8 µm, 2.1 mm × 100 mm).

Mobile phase = solvent A (pure water +0.1% formic acid) and solvent B (acetonitrile with 0.1% formic acid).

Gradient program = starting conditions of 95% A, 5% B. Within 9 min a linear gradient to 5% A, 95% B was programmed. A composition of 5% A, 95% B was kept for 60 s. Then a composition of 95% A and 5% B was adjusted for 70 s and maintained for 2.9 min.

Flow velocity = 0.3 ml/min.

Column oven = 40°C.

Injection volume = 4 µl.

The effluent was alternatively connected to an ESI-triple quadrupole-linear ion trap (QTRAP)-MS.

b. ESI-Q TRAP-MS/MS

We used triple quadrupole-linear ion trap mass spectrometer (Q TRAP, AB4500 Q TRAP UPLC/MS/MS) system to acquire LIT and QQQ (triple quadruple) scans. The system was equipped with ESI Turbo Ion-Spray interface and operated both in + and −modes. The system was controlled by Analyst 1.6.3 (AB Sciex) software. The parameters for ESI source operations were as follows.

Ion source, turbo spray; source temperature 550°C; ion spray voltage (IS) 5500 V (positive ion mode)/−4,500 V (negative ion mode); ion source gas I (GSI), gas II (GSII), curtain gas (CUR) were set at 50, 60, and 25.0 psi, respectively.

The collision-activated dissociation (CAD) was high. Instrument tuning and mass calibration were performed with 10 and 100 μmol/L polypropylene glycol solutions in QQQ and LIT modes, respectively. QQQ scans were acquired as MRM experiments with collision gas (nitrogen) set to medium. DP and CE for individual MRM transitions was done with further DP and CE optimization. A specific set of MRM transitions were monitored for each period according to the metabolites eluted within this period.

ii. Bioinformatic analyses

The metabolite normalized signal intensities were subjected to unit variance scaling before PCA in R by using prcomp function. Then we performed hierarchical cluster analysis (HCA) and represented it as heatmaps in R by using pheatmap. PCC was computed in R by using cor function.

Differentially accumulated metabolites (DAMs) between the Y, M, and O tissues were determined based on the following screening conditions i.e., VIP ≥1 and absolute Log2FC (fold change) ≥ 1. VIP values were extracted from OPLS-DA result, which also contain score plots and permutation plots, was generated using R package MetaboAnalystR. The data was log transform (log2) and mean centering before OPLS-DA. In order to avoid overfitting, a permutation test (200 permutations) was performed.

Metabolites were annotated in KEGG Compound database (http://www.kegg.jp/kegg/compound/) followed by mapping of the DAMs to KEGG Pathways database (http://www.kegg.jp/kegg/pathway.html). Pathways to which significantly regulated metabolites were mapped were then fed into MSEA (metabolite sets enrichment analysis), their significance was determined by hypergeometric test’s *p*-values.

## Conclusion

This three-stage ginger rhizome combined transcriptome and metabolome analyses revealed that the young tissues are transcriptionally and metabolically more active that the mature and older tissues. The key component of the ginger rhizome i.e., gingerol is present in higher quantities in the young tissues as compared to mature and old ones due to the higher expression levels of related genes. Similarly, the younger rhizome tissues were richer in linoleic acid and α-linoleic acid contents and the expression of the PLA2α and PLA2 homolog 3-like genes. On the contrary, the starch and sucrose biosynthesis was increased in mature and older ginger rhizomes tissues as compared to Y. The phytohormone signaling might be associated with the slowed growth in the mature and older ginger rhizomes. Taken together, this study proposes that the young ginger rhizome tissues are metabolically and transcriptionally more active and could be a preferred source of gingerol, linoleic acid, and α-linoleic acid.

## Data Availability

The datasets presented in this study can be found in online repositories. The names of the repository/repositories and accession number(s) can be found in the article/Supplementary Material.

## Glossary

4CL: 4-coumarate CoA ligase
ABA: abscisic acid
AHP: histidine-containing phosphotransfer protein
ARF: auxin responsive factor
AUX: auxin induced protein
BAHDb5a-1: BAHD family acyltransferase
BGLU22s: beta-glucosidase 22’s
C4H: cinnamate 4-hydroxylase
CCoAOMT: caffeoyl-CoA-O-methyltransferase
COMT: caffeic acid 3-O-methyltransferase
CS2: curcumin synthase 2
DAMs: differentially accumulated metabolites
DCSs: phenylpropanoylacetyl-CoA synthase
DEGs: differentially expressed genes
DPE: 4-alpha-glucanotransferase
EG: endoglucanase
FDR: false discovery rate
FPKM: fragments per kilobase of transcripts per million fragments mapped
GBSS: granule-bound starch synthase
HCT1s: hydroxycinnamoyltransferase 1
HST-like: shikimate O-hydroxycinnamoyltransferase-like
IAA: auxin responsive Protein
GH: indole 3-acetic acid amido synthetase
JA: jasmonic acid
JA-Ile: (-)-Jasmonoyl-L-Isoleucine
MAPK: mitogen activated protein kinases
Mature: M MS/MS
O: Old
OMT3: O-methyltransferase 3
PAL: phenylalanine ammonia lyase
PC: principal component
PCA: principal component analysis
PCC: Pearson’s correlation coefficients
PGM: phosphoglucomutase
PLA2α: phospholipase A2-alpha
POD: peroxidases
PYL: abscisic acid receptor
ROMTs: trans-resveratrol di-O-methyltransferase-like
SA: salicylic acid
SAPK: serine/threonine-protein kinase
SHT: spermidine hydroxycinnamoyl transferase
SPS: sucrose-phosphate synthases
UGP: UTP--glucose-1-phosphate uridylyltransferases (UGPs); UPLC
VSs: vinorine synthase-like
XET: xyloglucan endotransglucosylase/hydrolase proteins
Y: Young
