# Supplementary material for: Combined Metabolome and Transcriptome Analyses of Young, Mature, and Old Rhizome Tissues of Zingiber officinale Roscoe
Source: Front Genet. 2021 Dec 8;12:795201. doi: 10.3389/fgene.2021.795201 (PMC8692858; doi:10.3389/fgene.2021.795201)
Supplement: Supplementary file 1 [file DataSheet1.docx]

**Supplementary Figure 1.** Differential regulation of starch and sucrose metabolism pathway in ginger tuber. A) Y vs M, B) Y vs O, and C) M vs O. Where Y, M, and O represent young, mature, and old ginger tuber tissues, respectively.


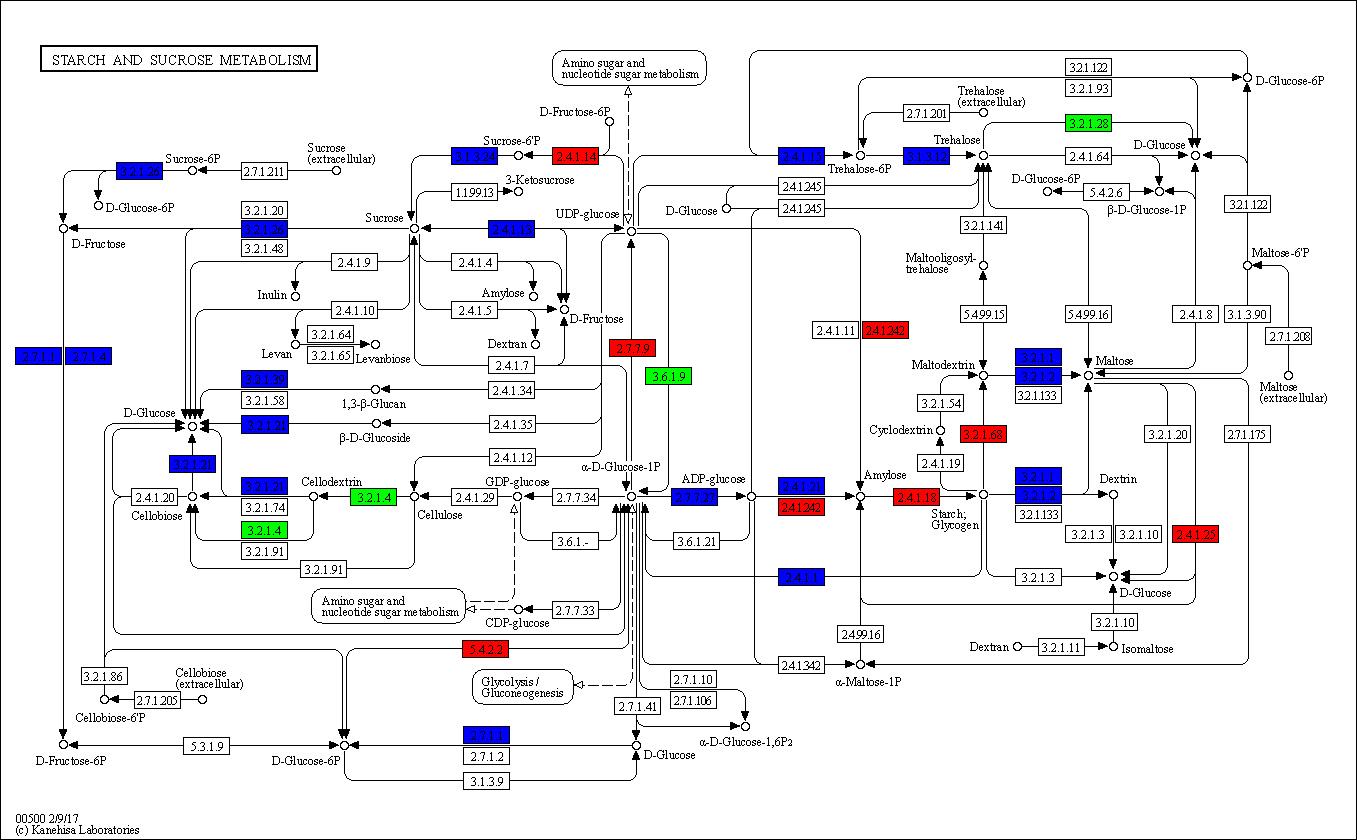


A


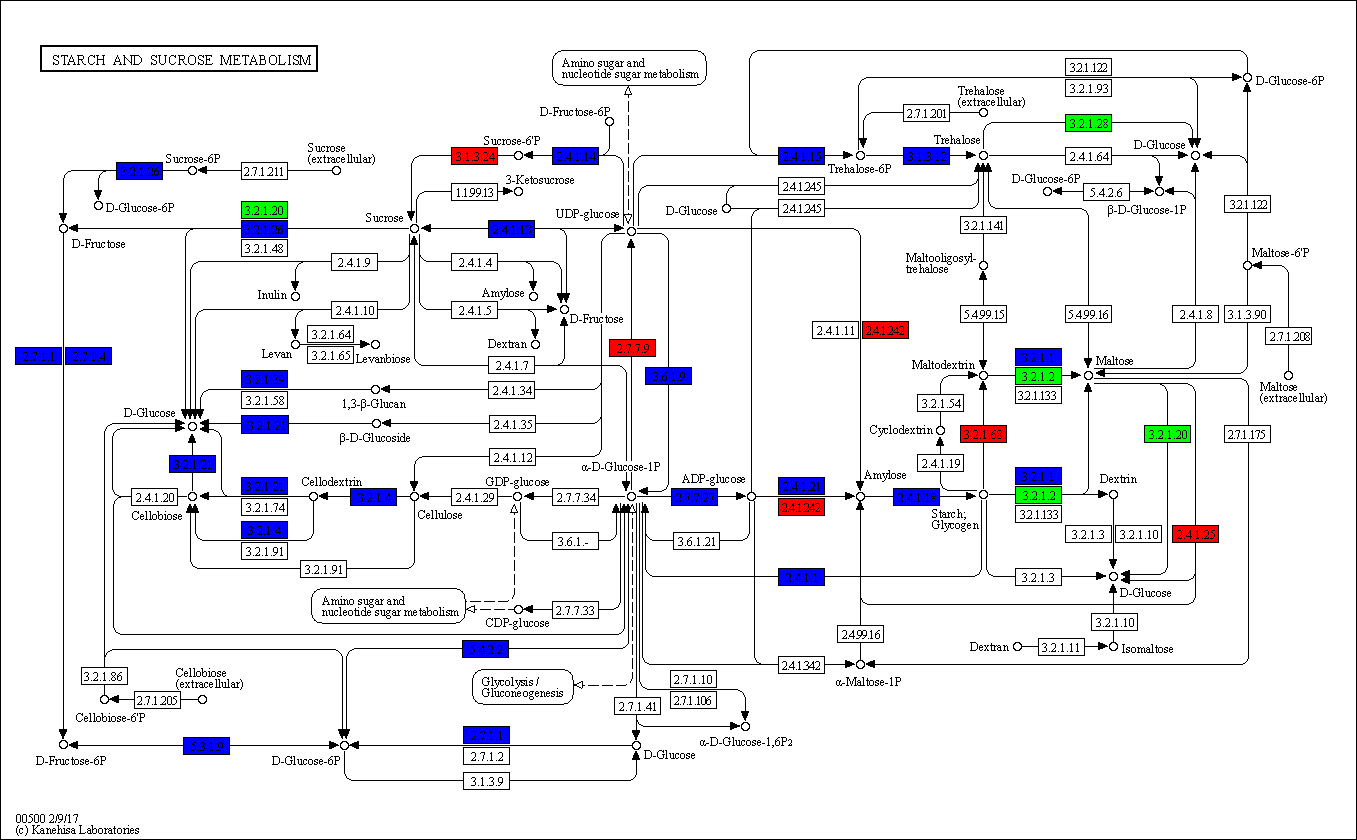


B


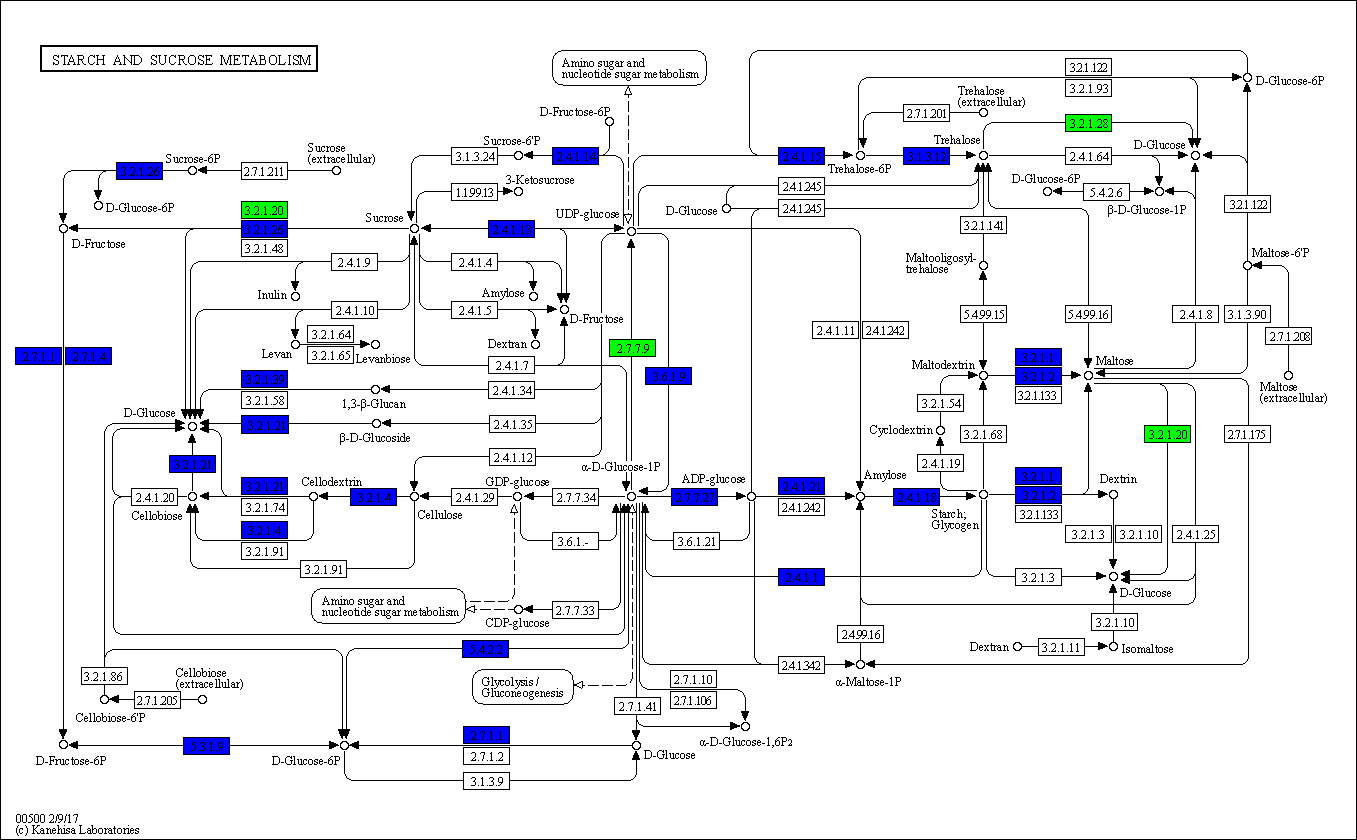


C

**Supplementary Figure 2.** Differential regulation of plant-hormone signaling pathway in ginger rhizomes. A) Y vs M, B) Y vs O, and C) M vs O. Where Y, M, and O represent young, mature, and old ginger tuber tissues, respectively.


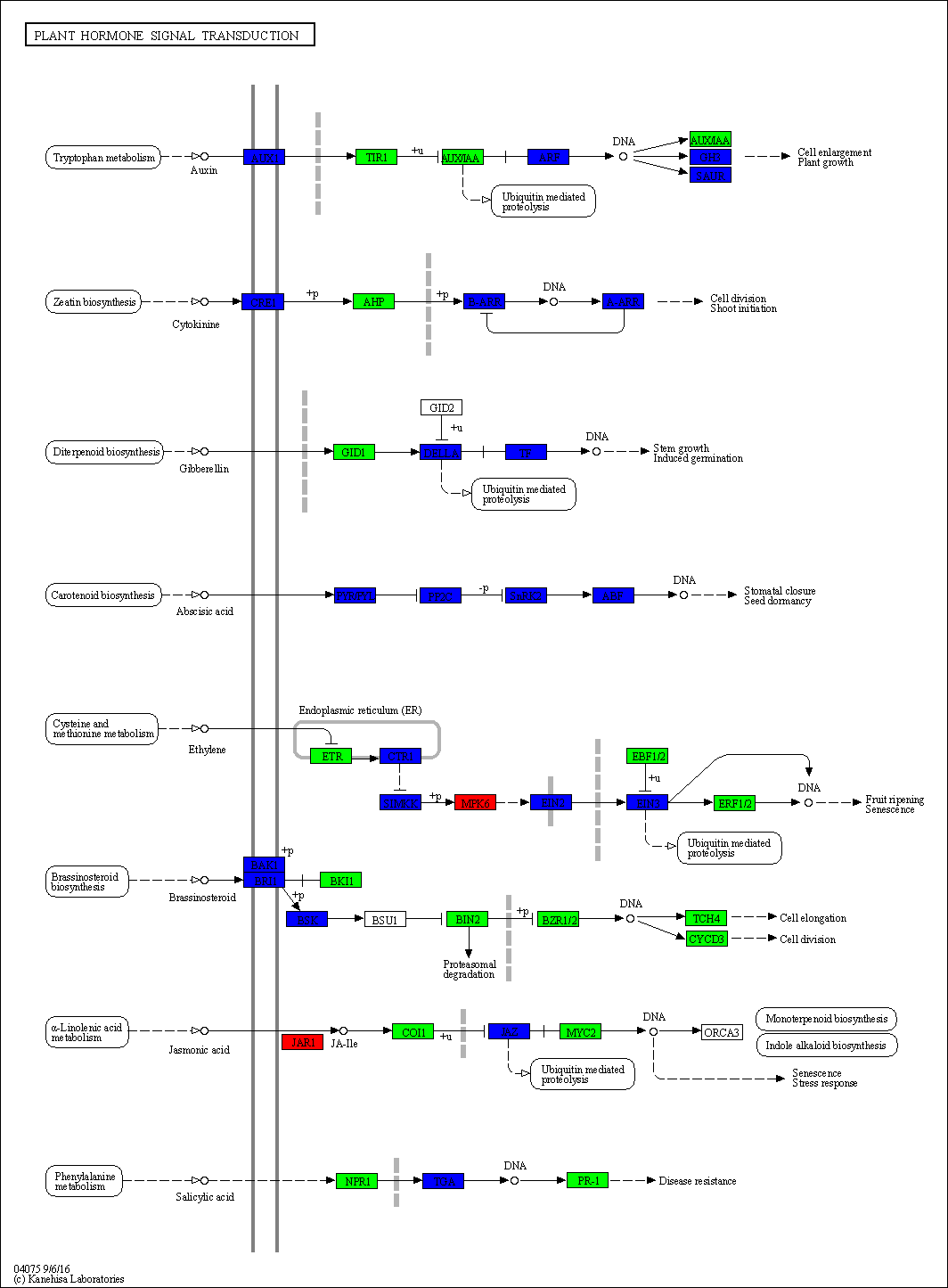


A


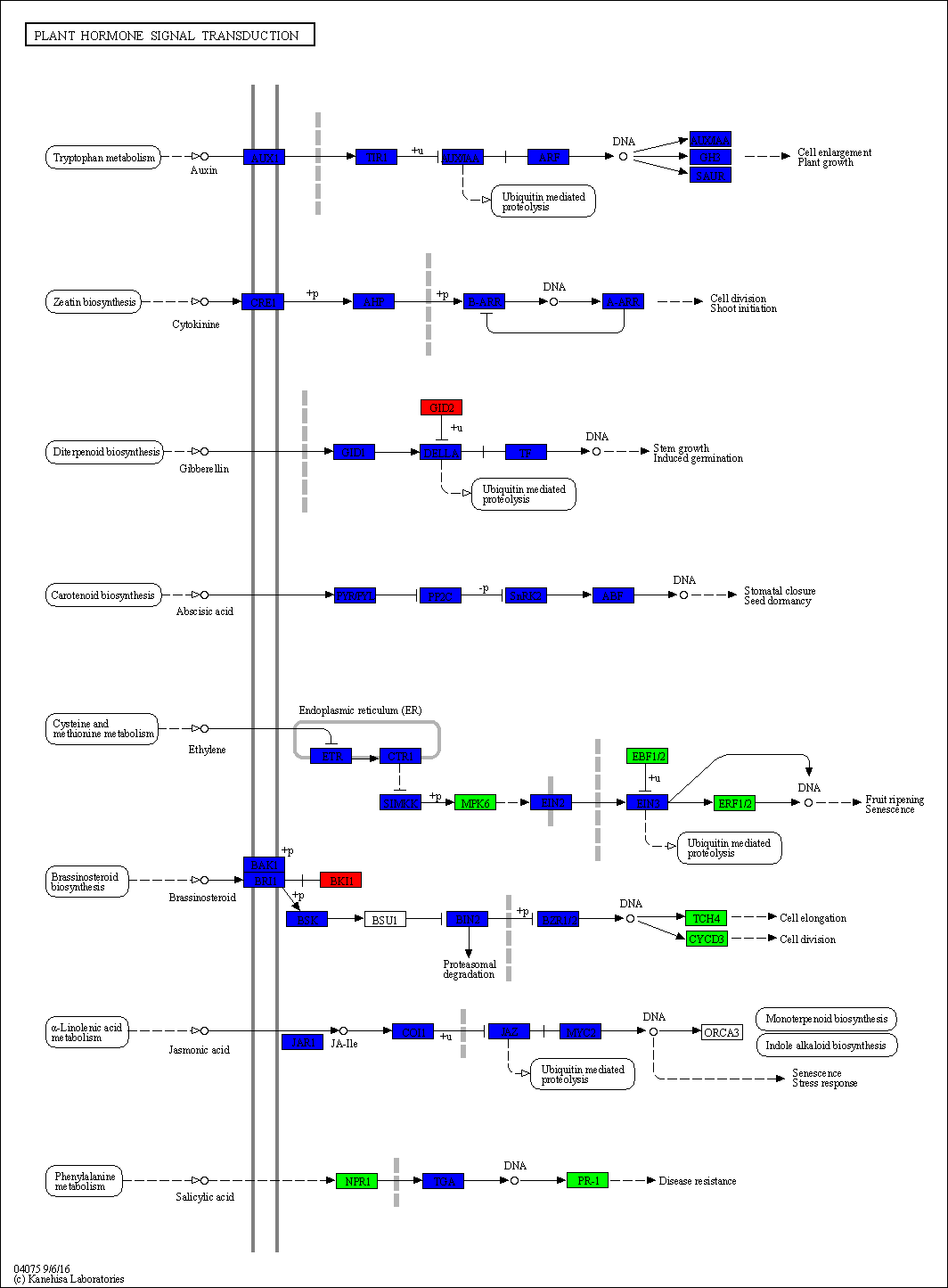


B


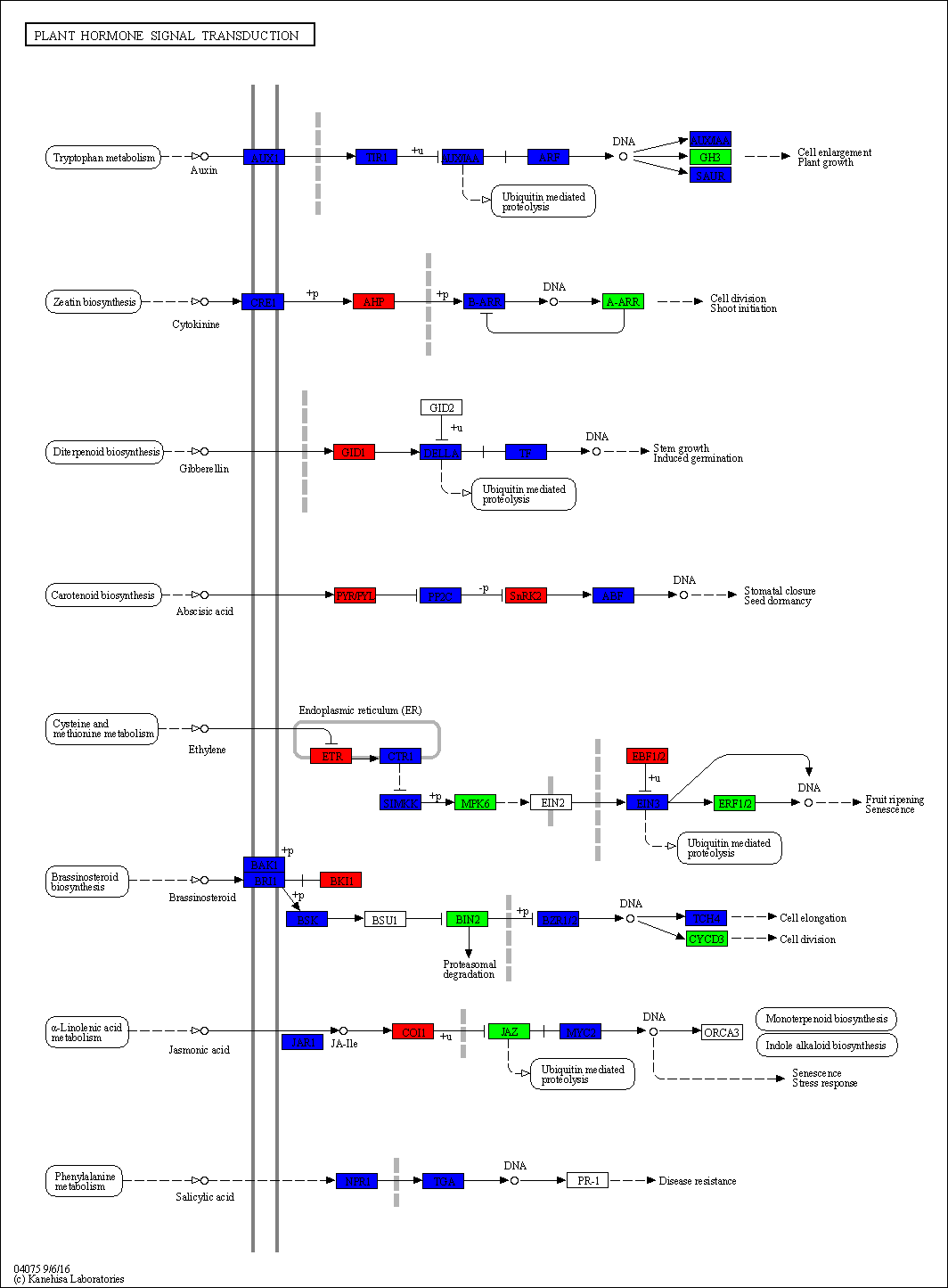


C
